# Metachromatic Leukodystrophy Presenting with Multiple Cranial Nerve and Lumbosacral Nerve Root Enhancement Without White Matter Changes

**DOI:** 10.3390/neurolint17020028

**Published:** 2025-02-16

**Authors:** Ruben Jauregui, Mekka R. Garcia, Thomas Mehuron, Steven L. Galetta, Devorah Segal

**Affiliations:** 1 Department of Neurology, NYU Grossman School of Medicine, 222 E 41st St, 14th Floor, New York, NY 10017, USA; garciam8@chop.edu (M.R.G.); steven.galetta@nyulangone.org (S.L.G.); devorah.segal@nyulangone.org (D.S.); 2 Department of Radiology, NYU Grossman School of Medicine, New York, NY 10017, USA; thomas.mehuron@nyulangone.org; 3 Department of Ophthalmology, NYU Grossman School of Medicine, New York, NY 10017, USA

**Keywords:** pediatric neurology, leukodystrophy, neuro-ophthalmology, neurogenetics

## Abstract

**Background**: Metachromatic leukodystrophy (MLD) is a rare autosomal recessive disorder that causes demyelination of both the central (CNS) and peripheral nervous systems (PNS). **Objective**: This study aims to report a unique MLD case presenting with cranial neuropathies and ataxia, initially without white matter changes on MRI, leading to diagnostic uncertainty. **Results**: A 20-month-old presented with bilateral abduction deficits, facial diplegia, and ataxia, raising the possibility of an acquired demyelinating condition. An MRI scan showed the enhancement of multiple cranial nerves, but normal white matter. A follow-up MRI showed new white matter changes that spared the U-fibers, suggesting a leukodystrophy. Biochemical assays were suggestive of metachromatic leukodystrophy, which was confirmed with genetic testing demonstrating a homozygous c.848+3A > G variant in *ARSA*. **Conclusions**: Our patient suggests that the initial presentation of MLD may mimic an acquired demyelinating condition and manifest with multiple cranial nerve palsies before more typical white matter changes evolve.

## 1. Introduction

Metachromatic leukodystrophy (MLD) is a rare autosomal recessive disorder belonging to the group of lysosomal storage diseases [1]. It is caused by pathogenic variants in the gene *ARSA*, which encodes for the enzyme arylsulfatase A (ARSA) [1]. Deficient activity of ARSA leads to the accumulation of sulfatides in neural tissues, causing demyelination of both the central (CNS) and peripheral nervous systems (PNS) [1]. The mechanism behind demyelination in MLD is unclear, but studies have suggested that an increase in sulfatides may lead to instability of the myelin sheath, while elevated sulfatides also cause calcium to accumulate in the cell cytoplasm, leading to cell stress and apoptosis [1,2]. The accumulation of sulfatides may also trigger an inflammatory response, as the elevation of various cytokines has been reported in the cerebrospinal fluid and serum of MLD patients [3].

MLD is classified into three clinical forms based on the age of first symptom onset, namely late-infantile (<30 months), adult (>16 years), and juvenile (30 months–16 years), though the latter can also be divided into early-juvenile (30 months–6 years) and late-juvenile (6 years–16 years) [1,4]. The late-infantile form is the most common, accounting for 50–60% of MLD cases worldwide, whereas 20–30% of patients are diagnosed with the juvenile type, and 15–20% of patients with the adult type [1]. Generally, symptom onset at an earlier age is associated with a more severe and rapid disease progression [1,4]. Considered to be the most severe, the late-infantile form typically presents with symptoms including gait abnormalities, a delay in motor milestones, and the regression of speech, cognitive, and motor functions, while signs of peripheral neuropathy and weakness are also present [1]. The terminal stage is additionally associated with the development of psychomotor retardation and bulbar and pseudobulbar palsy complicated by difficulties in swallowing and breathing, with disease management focused on palliative care [1,5]. The rapid and severe course of the disease in the late-infantile form often causes death in childhood [1,5]. Compared to the early MLD forms (late-infantile and early-juvenile), the late-juvenile and adult forms present with a less severe disease course, characterized by early cognitive, behavioral, and psychiatric symptoms, with motor regression occurring later in the disease [1,4,5].

Diagnosis is based on a constellation of clinical manifestations, genetic testing, imaging, and biochemical assays. Genetic testing is the most specific method for diagnosis, but in cases where pathogenetic variants cannot be identified or testing is not available, biochemical assays can help in establishing a diagnosis [1,4]. These assays measure sulfatides in the urine and the level of ARSA enzymatic activity. Of note, ARSA pseudo-deficiency, a condition where the enzymatic activity of ARSA is in the range of MLD patients, can occur in individuals, but they will remain asymptomatic throughout life and have negligible to no sulfatide accumulation in the urine [4]. Magnetic resonance imaging (MRI) of the brain in MLD shows symmetric white matter involvement sparing the subcortical U-fibers [1,6]. In advanced cases, the presence of a “tigroid pattern” can be observed due to preservation of myelin in perivenular areas [1,6]. U-fiber involvement is seen in the late stages and is associated with severe motor dysfunction [6].

We present an atypical case of MLD, where the patient presented with facial diplegia and bilateral eye abduction deficits. The initial MRI only revealed isolated multiple cranial nerve and lumbosacral nerve root enhancement, eventually progressing to develop intraparenchymal white matter involvement typically associated with leukodystrophies on subsequent imaging.

## 2. Case Description

A previously healthy 20-month-old male with no medical history was referred to the child neurology clinic for the evaluation of esotropia and abnormal MRI brain results. He was born at 36 weeks via non-spontaneous vaginal delivery from a non-complicated pregnancy. Newborn screening, which included Krabbe disease, was negative. Family history was notable for a healthy older sister, and otherwise noncontributory. The patient was of Sephardic Jewish descent. At the time of presentation, he was meeting his developmental milestones.

**Figure 1:**
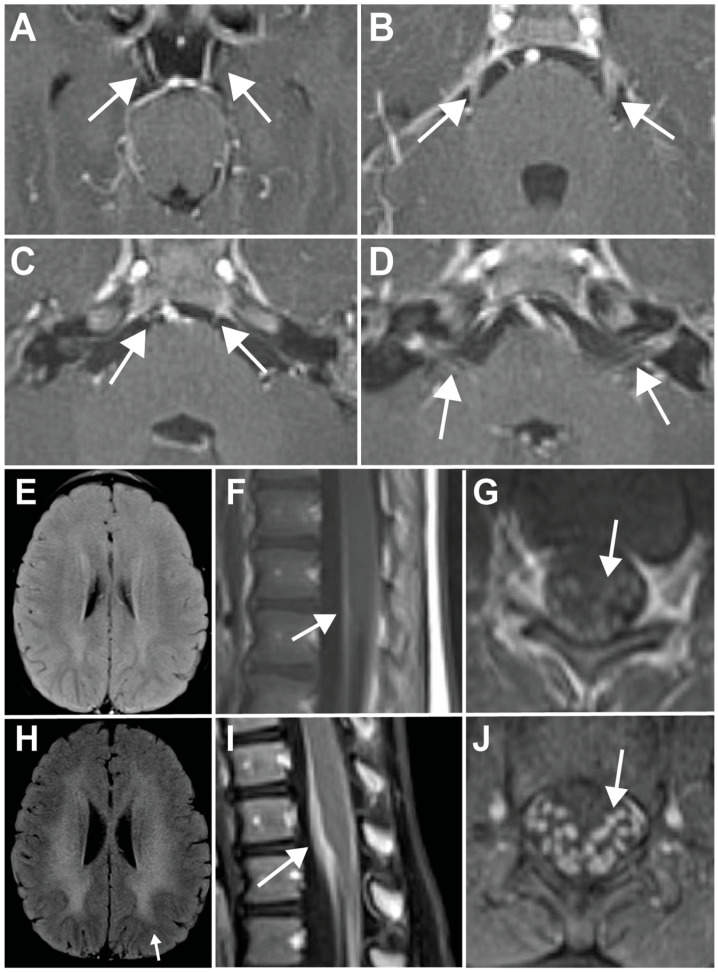
Magnetic resonance imaging (MRI) of the brain demonstrating the abnormal enhancement of the oculomotor (**A**), trigeminal (**B**), abducens (**C**), and facial and vestibulocochlear (**D**) cranial nerves (arrows). On presentation, MRI of the brain did not reveal signal abnormalities in the cerebral white matter (**E**), while MRI of the spine revealed the abnormal enhancement of the thecal sac (sagittal view, (**F**)) and lumbosacral nerve roots (axial view, (**G**)). Five months later, a repeat MRI of the brain was now notable for confluent white matter hyperintensities sparing the subcortical U-fibers (arrow), demonstrating a rapid progression of white matter disease (**H**), while the enhancement of the thecal sac (**I**) and nerve roots (**J**) was more pronounced.

The mother had noted the patient to be “cross-eyed” 4 months prior, which led to a referral to pediatric ophthalmology. He was initially diagnosed with alternating esotropia and was prescribed an eye patch. There was no improvement in his esotropia, and he was then evaluated with an MRI of the brain before undergoing corrective strabismus surgery. MRI of the brain revealed enhancement of multiple bilateral cranial nerves, including III, V, VI, VII, and VIII, with no significant white matter changes observed at that time (Figure 1A–E), leading to a referral to child neurology.

On evaluation, the mother also reported that he drooled excessively and was “extremely clumsy”, leading to multiple falls. The neurologic exam was notable for bilateral eye abduction deficits and symmetric bilateral lower face diplegia with flat nasolabial folds. There was no focal weakness, reflexes were 2+ throughout, and there were bilateral extensor plantar responses. The patient was uncoordinated when reaching for objects, with a wobbling gait and frequent falling. An MRI of the total spine was also obtained and revealed leptomeningeal enhancement along the thecal sac (Figure 1F,G). Cerebrospinal fluid (CSF) studies from a lumbar puncture were normal, including protein, glucose, cell count, and infectious studies. An electromyography (EMG) study demonstrated a length-dependent, asymmetric, demyelinating sensorimotor polyneuropathy. A subsequent extensive infectious, auto-immune, and paraneoplastic work-up was unrevealing, including GQ1b antibodies to evaluate for etiologies such as Miller Fisher syndrome. Over the subsequent months, the patient continued to be ataxic and had worsening bilateral leg weakness and pain.

On examination 5 months later, there was improvement of the bilateral abduction deficits, but the facial diplegia persisted. Bilateral foot drop with steppage gait was noticed, along with hyporeflexia throughout. He had a wide-based gait and required support to walk. At this time, the work-up with MRI and lumbar puncture was repeated. CSF studies were only notable for a mild elevation of protein to 65 mg/dL [normal range: 15–60 mg/dL]. An MRI of the total spine revealed increased leptomeningeal enhancement of the thecal sac and cauda equina nerve roots (Figure 1I,J). Notably, MRI of the brain revealed new diffuse periventricular white matter abnormalities, along with the persistent bilateral enhancement of multiple cranial nerves that was previously observed (Figure 1H). Given the extensive white matter changes seen on MRI, the patient underwent a work-up for leukodystrophies, particularly metachromatic leukodystrophy (MLD), given the CNS and PNS findings. The patient was found to have decreased ARSA enzyme activity (35 nmol/h/mg, normal reference range >62 nmol/h/mg) and elevated urine sulfatides, suggestive of a diagnosis of MLD. Genetic testing revealed a homozygous c.848+3A > G (g.51065016) variant in the *ARSA* gene (GenBank NM_000487)*,* classified as a variant of uncertain significance as per the ACMG criteria [7]. Given the adjuvant biochemical assays demonstrating decreased ARSA enzymatic activity and elevated urine sulfatides, along with the clinical presentation, this variant was determined to be causative and the diagnosis of MLD was confirmed. The patient was referred to a leukodystrophy center for treatment evaluation. Based on the available treatments at the time, the plan was made for the patient to undergo bone marrow transplantation (BMT). Unfortunately, the patient expired during the conditioning regimen.

## 3. Discussion

MLD typically presents with extensive white matter abnormalities on MRI with characteristic sparing of the subcortical U-fibers [8]. Although rare, there are few reported cases in the literature where multiple cranial nerve enhancement is seen in MLD [8,9,10]. Similarly to our patient, those previously reported cases presented with decreased reflexes and varying degrees of cranial neuropathies in the setting of multiple cranial nerves enhancement [8,9,10]. The patient described by Singh et al. presented with bilateral facial weakness and ocular motor abnormalities (lateral rectus palsy), whereas the patient in Sonowal et al. also had ocular motor abnormalities (horizontal nystagmus), while the patient in Maia et al. had an unremarkable cranial nerve examination [8,9,10]. In contrast to our patient, however, the patients described in Maia et al. and Sonowal et al. had extensive white matter abnormalities on MRI at the time of presentation [8,9]. On the initial MRI for our patient, there was enhancement of multiple cranial nerves without any significant white matter abnormalities on the MRI. Singh et al. also reported a similar case to ours with isolated cranial nerve enhancement without white matter involvement on presentation [10]. Regarding our patient’s abduction deficits, acute strabismus has been reported as a potential early sign of late-infantile MLD by Beerepoot et al. [11]. In a cohort of 204 MLD patients, the development of strabismus before motor symptoms was seen exclusively in patients with late-infantile MLD [11]. Furthermore, 27% of late-infantile MLD patients developed strabismus, and among those with an MRI close to the onset of strabismus, 46% presented with no white matter abnormalities on MRI, just cranial nerve enhancement [11]. The lack of white matter involvement in the setting of cranial nerve enhancement and evidence of peripheral polyneuropathy creates a diagnostic challenge for clinicians. In this scenario, a leukodystrophy would be less likely to be suspected in the absence of other suggestive factors such as family history, leading the clinician to consider and work-up for other etiologies, such as Guillain–Barre or Miller Fisher syndrome. Thus, only when the MRI of the brain was repeated 5 months later and revealed more extensive white matter disease did the team pursue a work-up to evaluate for MLD.

MLD is one of the few leukodystrophies that affects both the CNS and PNS, as the accumulation of sulfatides causes demyelination in both [12]. In our patient, the initial presentation was indicative of a process affecting the PNS, with EMG demonstrating a demyelinating sensorimotor polyneuropathy and MRI of the brain/total spine revealing multiple enhancing cranial nerves and lumbosacral nerve roots. Only when extensive CNS white matter changes developed later did it become evident that the CNS was also affected, causing the team to pursue targeted testing for MLD specifically, rather than a broader leukodystrophy evaluation. Other leukodystrophies associated with involvement of both CNS and PNS include adrenoleukodystrophy and Krabbe disease, which are widely included in newborn screens and thus accelerates their diagnosis when patients become symptomatic [1,12]. Furthermore, enlargement of the optic nerves and chiasm due to the accumulation of globoid cells has also been reported in Krabbe disease [13,14,15]. Given the clinical overlap between MLD and Krabbe with the potential involvement of both the CNS and PNS, this neuroimaging finding can guide clinicians in their evaluation, particularly for patients that have not had a prior newborn screen or genetic testing.

Despite being a leukodystrophy, our case demonstrates that MLD can initially present with isolated cranial nerve enhancement on MRI. As noted in our patient, white matter abnormalities eventually develop over time, but an earlier diagnosis could have been made if MLD had been considered with the first MRI and subsequently an earlier referral to a treatment center. BMT and allogeneic hematopoietic stem cell transplants (HSCT) have been used for the treatment of MLD [16]. Overall, the outcomes from BMT and HSCT have been mixed, generally demonstrating limited resultsin patients with early-onset MLD or those with advanced symptoms, while better results are seen for those with late-juvenile or adult-onset MLD [16,17,18,19,20,21,22]. For example, a study of juvenile MLD patients demonstrated improved motor and language outcomes along with lower MRI severity scores after undergoing HSCT [17]. Yet, a systematic review of the literature on the clinical effectiveness of different currently available treatments reported no clinical benefits (survival, gross motor and cognitive function) for late-infantile MLD patients that received HSCT [16]. This contrasts with the late-infantile MLD patients that received arsa-cel gene therapy, where improvement in the aforementioned clinical markers was seen [16]. Arsa-cel is a form of gene therapy that contains autologous hematopoietic stem and progenitor cell populations transduced ex vivo with a lentiviral vector encoding human ARSA cDNA [5]. In a clinical trial with 26 pre-symptomatic or early-symptomatic pediatric patients with early-onset MLD (late-infantile and early-juvenile types, age <7 years at disease onset), Fumagalli et al. reported that treatment with arsa-cel led to preserved cognitive function and motor development in most patients, while slowing demyelination and brain atrophy [5]. Given the success and promise of arsa-cel therapy, a timely diagnosis is crucial so that MLD patients may receive beneficial treatments. At the time of the referral for our reported patient, BMT was the only available treatment, but nowadays, he would have likely received arsa-cel therapy given his late-infantile MLD. Both European and U.S. consensus guidelines recommend referral to a treatment center for evaluation and arsa-cel therapy for late-infantile patients [4,23].

The onset of new treatment modalities has spurred the movement for MLD to be included in newborn screens. For example, at the time our reported patient was born, his newborn screen included Krabbe disease but not MLD. A study by Bean et al. reported that the implementation of newborn screening for MLD in the United Kingdom would be cost-effective, as it would accelerate the time for the diagnosis and treatment of patients while potentially decreasing their future disability from MLD and the burden on the resources of the healthcare system [24]. In addition, Laugwitz et al. reported the success of a newborn screening program in Germany in order to treat pre-symptomatic newborns [25]. Two of these patients were predicted to have early-onset MLD based on newborn screening and received arsa-cel therapy at the age of 12 months, while one was predicted to have late-onset MLD and was planned to undergo allogenic HSCT treatment at a later age [25]. By the time of the study, it was reported that these three patients were meeting their developmental milestones after receiving treatment [25]. Newborn screening for MLD is also supported by consensus studies on MLD recommendations and clinical management from both Europe and the United States [4,23].

## 4. Conclusions

When a patient presents with multiple enhancing cranial nerves, MLD should be considered in the differential even in the absence of white matter changes on an MRI of the brain. Additionally, during evaluation for leukodystrophies, the evidence of additional PNS involvement in addition to CNS is highly specific for MLD. Given the encouraging results of gene therapy in pre-symptomatic/early-symptomatic early-onset MLD patients and HSCT for juvenile MLD patients, recognizing and establishing a diagnosis in a timely manner is crucial so that patients may benefit from these therapies. Furthermore, with the onset of these therapies, there is a proposed movement to include MLD in newborn screening programs to accelerate the identification of these patients and potentially treat them even before they become symptomatic.

## Data Availability

The original contributions presented in the study are included in the article, further inquiries can be directed to the corresponding author/s.
